# Parameter Calibration of Rotating Wave Plate Polarization Detection Device Using Dual Beams

**DOI:** 10.3390/s25154803

**Published:** 2025-08-05

**Authors:** Haonan Zhang, Junbo Liu, Ziliang Yan, Chuan Jin, Jian Wang, Song Hu

**Affiliations:** 1 University of Chinese Academy of Sciences, Beijing 100049, China; zhanghaonan23@mails.ucas.ac.cn; 2 Institute of Optics and Electronics Chinese Academy of Sciences, Chengdu 610209, China; liujunbo@ioe.ac.cn (J.L.); yanziliang2092@foxmail.com (Z.Y.); wangjian@ioe.ac.cn (J.W.); husong@ioe.ac.cn (S.H.)

**Keywords:** Stokes parameter, rotating wave plate method, polarization detection device, parameter calibration

## Abstract

When measuring Stokes parameters using the rotating wave plate method, the angle error of the polarizer’s light transmission axis, the azimuth error of the wave plate’s fast axis, and the phase delay error are key factors restricting accuracy. To address the existing calibration methods’ insufficient accuracy and incomplete consideration of the error parameters, this study constructed an error-transfer analytical model for an in-depth analysis of the principle of measuring Stokes parameters using the rotating wave plate method. It also clarified the quantitative parameter relationship between the measurement, wave plate, and polarizer errors. A device parameter calibration scheme using multi-angle polarized light (horizontally linearly polarized, [1,1,0,0]^T^, and 45° linearly polarized, [1,0,1,0]^T^) was further proposed, and by using the deviation between the theoretical response of the standard incident light and the actual measurement data, an error equation was established to solve the device parameter error and precisely calibrate the polarization detection device. The experimental results show that after using this method, the calibration error of the Stokes parameters decreased from 4.83% to within 0.46%, significantly overcoming the traditional methods’ limitations regarding incomplete consideration of the error parameters and accuracy improvement, providing a more concise and reliable method for high-precision polarization measurement.

## 1. Introduction

**Figure 1:**
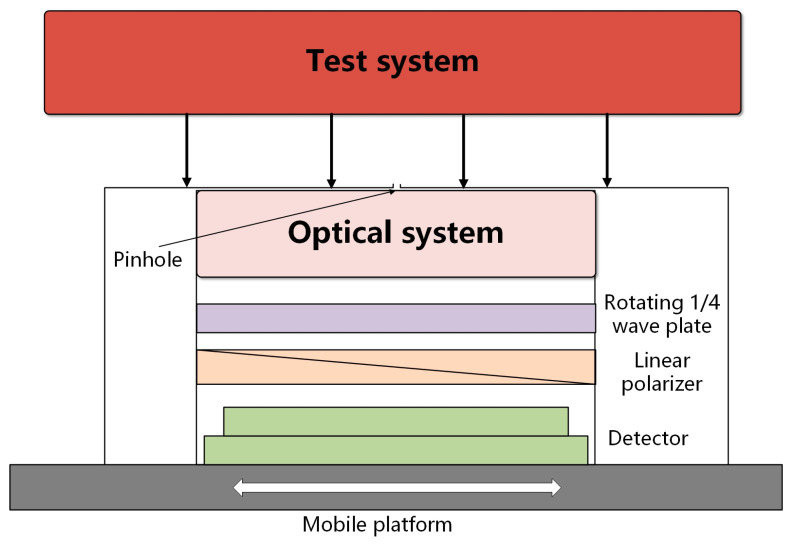
Schematic diagram of the polarization sensor.

Integrated circuits (ICs) have been foundational in the development of all current high-tech fields [1]. The degree of IC integration keeps increasing in accordance with Moore’s law, and photolithography technology is at the core of manufacturing very large-scale ICs [2,3]. With an increase in the numerical aperture of an optical lens system, the polarization of the illumination beam has a substantial effect on the lithography imaging quality [4,5]. The adoption of polarized and off-axis illumination can greatly enhance the image contrast of a photolithography system [6,7]. Correspondingly, photolithography systems also have high performance requirements for the illuminated polarized light. High-precision detection of polarization parameters is a prerequisite for effectively regulating them in a photolithography exposure system [8,9]. The rotating wave plate method [10] achieves optical modulation by rotating a 1/4 wave plate and fixed polarizer and uses optical imaging devices and photodetectors for signal acquisition. When using this method, it is necessary to measure the polarization states of various illumination modes incident on the mask surface. The principle of pinhole imaging can be utilized. A small hole is made in the mask surface and then connected to a lens; this forms an image of the illumination light converging on the surface for the rear detector to measure. Figure 1 shows a schematic diagram of the polarization sensor developed for use in photolithography.

Three quantitative parameter errors—the angle error of the polarizer’s light transmission axis, the azimuth error of the 1/4 wave plate’s fast axis, and the phase delay error—are the main factors affecting the measurement accuracy of rotating wave plate-based Stokes polarization detection devices used in imaging [11,12,13,14]. By calibrating the device parameters, the measurement accuracy can be effectively improved.

The main techniques used for detecting the Stokes parameters of light sources include the polarized light modulation and partial amplitude methods [15,16]. The former is suitable for measuring stable and continuous light waves, while the latter is generally applied for measuring the polarization state of pulsed light beams. The four-point [17,18] and equator–poles (E-P) calibration methods [19,20] are adopted in the vast majority of amplitude division light polarizers at present [21]. The four-point calibration method is relatively simple, but it provides less information and has a large systematic error, making it difficult to obtain a true instrument matrix. The E-P method measures many polarization states and provides a large amount of information, but it is rather complex. Foreign countries became involved in this field earlier, and their technologies are relatively mature. Many measurement and calibration technologies have been commercialized. However, the published research on this topic is relatively limited both domestically and internationally, with the primary contribution being the optical modulation-based online detection and calibration technology developed by the Toshiba Nomura team [22,23,24]. Their detection system utilizes an independently fabricated polarization mask to measure the projection objective lens’s Mueller pupil. However, a significant drawback of this approach is the inherent difficulty and high cost associated with fabricating the polarization mask. At present, domestic technology is relatively underdeveloped, and its measurement accuracy is generally low. The Stokes polarizer error calibration and compensation method proposed by Li Yanqiu’s team from the Beijing Institute of Technology [11] considers the wave plate’s fast-axis angle error and phase delay error but fails to take into account the polarizer’s transmission–axis angle error. The device parameter calibration method proposed by Bu Yang’s team from the Shanghai Institute of Optics and Fine Mechanics, the Chinese Academy of Sciences [13], requires the rotation of the polarizer’s azimuth angle, which introduces an angle error in the polarizer’s light transmission axis. In his doctoral dissertation, Du Xiliang [25] from the Harbin Institute of Technology employed the E-P calibration method to mitigate the influence of defects in the polarization state generator’s optical components on the instrument matrix. However, this necessitates the production of at least four linearly independent standard reference polarization states using the generator, and each reference state must undergo a minimum of four measurements. Consequently, the calibration procedure is notably complex.

To address the limitations of the existing methods, including their insufficient accuracy, complex calibration procedures, and inadequate consideration of the error parameters, this paper proposes a system parameter error calibration scheme for the measurement system based on Fourier analysis [26,27,28] and the rotating wave plate method. Focusing on the demand for high-precision polarization measurement and considering the core multi-dimensional quantitative parameter errors, such as the angle error of the light transmission axis of the polarizer, the angle error of the fast axis of the wave plate, and the phase delay error of the wave plate, a “dual standard polarized light calibration” strategy was designed, using 0- and 45-line polarized light as calibration sources. The essence of this strategy lies in leveraging the minimal optimal basis of dual beams to capture the key measurement characteristics associated with the degrees of freedom. Specifically, the orthogonality of 0° and 45° linearly polarized light within the S1–S2 plane allows for the direct construction of an equation system encompassing the coupling relationships among all the parameters to be calibrated, using only two measurements. This approach facilitates the precise calibration of a polarization detection device. The simulation and experimental results show that this scheme can significantly reduce the error interference and improve the measurement accuracy.

## 2. Basic Principles and Methods

### 2.1. The Basic Principles of the Rotating Wave Plate Method

**Figure 2:**
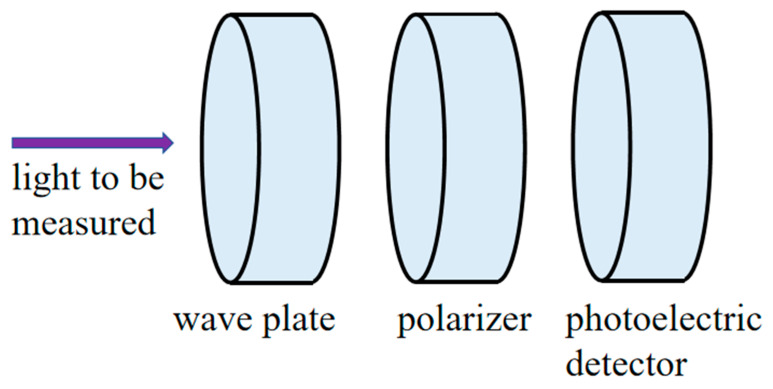
Schematic diagram of the rotating wave plate method.

The rotating wave plate method is a full Stokes polarization measurement technique. Its core principle lies in using a rotatable quarter wave plate to periodically adjust the polarization state of the incident light to be measured. Then, using a fixed-direction polarizer (usually a linear polarizer), the modulated light intensity signal is converted into an electrical signal that varies over time. Finally, four Stokes parameters containing all the incident light’s polarization information are extracted through Fourier analysis of this signal. Figure 2 is a schematic diagram of the rotating wave plate method, with the setup composed of a 1/4 wave plate with a rotating motor, a fixed polarizer, an optical imaging device, and a CCD. This method is usually used to measure a polarized illumination light source. The incident light beam passes successively through the rotatable 1/4 wave plate and fixed polarizer and then is imaged on the CCD’s photosensitive surface using the optical imaging device. Finally, the CCD completes the image acquisition.

The Stokes vector of the incident light is S = [S_0_,S_1_,S_2_,S_3_]^T^. When the rotation angle is θ, the Muller matrix of the 1/4 wave plate is

(1) $$Q_{\theta }=\left[\begin{array}{cccc} 1 & 0 & 0 & 0\\ 0 & \cos^{2}2\theta & \sin2\theta \cos2\theta & -\sin2\theta \\ 0 & \sin2\theta \cos2\theta & \sin^{2}2\theta & \cos2\theta \\ 0 & \sin2\theta & -\cos2\theta & 0 \end{array}\right]$$

The Muller matrix of an ideal linear polarizer with an angle of θ between the transmission and horizontal axes is

(2) $$P=\frac{1}{2}\left[\begin{array}{cccc} 1 & \cos2\theta & \sin2\theta & 0\\ \cos2\theta & \cos^{2}2\theta & \sin2\theta \cos2\theta & 0\\ \sin2\theta & \sin2\theta \cos2\theta & \sin^{2}2\theta & 0\\ 0 & 0 & 0 & 0 \end{array}\right]$$

Assuming that the optical imaging device does not affect the polarization state of the light beam, then, according to Muller matrix theory, the Stokes vector of the emergent light, S′, is

(3) $$S^{\prime }=P\cdot Q_{\theta }\cdot S$$

The light intensity detected by the detector, I(θ) = S_0_′, is

(4) $$I(\theta )=\frac{1}{2}\left[\left(S_{0}+\frac{S_{1}}{2}\right)+\frac{S_{1}}{2}\cos4\theta +\frac{S_{2}}{2}\cos4\theta -S_{3}\sin2\theta \right]$$

Substituting θ = ωt, we can write this as

(5) $$I(\omega t)=\frac{1}{2}\left[A-B\sin2\omega t+C\cos4\omega t+\mathrm{Dsin}4\omega t\right]$$

In the above formula, A, B, C, and D are, respectively,

(6) $$\left\{\begin{array}{l} A=S_{0}+\frac{S_{1}}{2}=\frac{1}{\pi }\int_{0}^{2\pi }I(\theta )d\theta \\ B=S_{3}=\frac{2}{\pi }\int_{0}^{2\pi }I(\theta )\sin2\theta d\theta \\ C=\frac{S_{1}}{2}=\frac{2}{\pi }\int_{0}^{2\pi }I(\theta )\cos4\theta d\theta \\ D=\frac{S_{2}}{2}=\frac{2}{\pi }\int_{0}^{2\pi }I(\theta )\sin4\theta d\theta \end{array}\right.$$

The above derivation process does not take into account the parameter errors of the Stokes polarization imaging device, while the ideal polarization device does not exist in practical applications. Therefore, the parameter errors need to be taken into consideration. The specific values of each are determined through error calibration to achieve error compensation.

### 2.2. Principles of Error Calibration and Compensation

Suppose that the angle error of the polarizer’s light transmission axis is α, the fast-axis angle error of the 1/4 wave plate is ∆θ, and the phase delay value error is δ. The phase retardation of a quarter wave plate is ideally 90, whereas it is actually 90° + δ. Considering the angular error of the 1/4 wave plate’s fast axis, δ, and the phase delay error, θ, when the rotation angle is q, let θ′ = θ + ∆θ; then, its Muller matrix is

(7) $$Q^{\prime }=\left[\begin{array}{cccc} 1 & 0 & 0 & 0\\ 0 & \cos^{2}2\theta^{\prime }-\sin\delta \sin^{2}2\theta^{\prime } & (1+\sin\delta )\sin2\theta^{\prime }\cos2\theta^{\prime } & -\cos\delta \sin2\theta^{\prime }\\ 0 & (1+\sin\delta )\sin2\theta^{\prime }\cos2\theta^{\prime } & \sin^{2}2\theta^{\prime }-\sin\delta \cos^{2}2\theta^{\prime } & \cos\delta \cos2\theta^{\prime }\\ 0 & \cos\delta \sin2\theta^{\prime } & -\cos\delta \cos2\theta^{\prime } & -\sin\delta \end{array}\right]$$

The light intensity value detected by the CCD is

(8) $$I(\theta_{i})=(1,0,0,0)\cdot P(\alpha )\cdot Q\frac{\pi }{2}+\delta (\theta_{i}+\Delta \theta )\cdot S_{0}$$

The coefficients A, B, C, and D are related to the error parameters and incident light Stokes vector as follows:

(9) $$\left\{\begin{array}{c} A=S_{0}+\frac{1}{2}(1-\sin\delta )\left(S_{1}\cos2\alpha +S_{2}\sin2\alpha \right)\\ B=\cos\delta \left(S_{1}\sin2\alpha -S_{2}\cos2\alpha \right)\\ C=\frac{1}{2}(1+\sin\delta )\left(S_{1}\cos(2\alpha -4\Delta \theta )-S_{2}\sin(2\alpha -4\Delta \theta )\right)\\ D=\frac{1}{2}(1+\sin\delta )\left(S_{1}\sin(2\alpha -4\Delta \theta )+S_{2}\cos(2\alpha -4\Delta \theta )\right) \end{array}\right.$$

To accurately calibrate the polarization response of the system and calculate the error parameters, we selected 0° and 45° linearly polarized light sources as characteristic references. The 0° linearly polarized light corresponds to the normalized Stokes vector S0 = [1,1,0,0]^T^, and its electric field vibrates along the horizontal axis, allowing us to precisely calibrate the response of the system to the horizontal polarization component. The 45° linearly polarized light corresponds to the normalized Stokes vector S1 = [1,0,1,0]^T^, and the electric field vibrates at a 45° angle to the horizontal axis, which is used to characterize the system’s response to the oblique polarization component. The orthogonality of the 0° and 45° linearly polarized light in the S1–S2 plane causes the originally coupled S1 and S2 terms to manifest separately within two distinct sets of equations. Consequently, the problem of solving for multiple parameter errors is reduced to a dual-dimensional constraint defined by the “horizontal polarization response equation” and the “45° polarization response equation”. Due to this dimensional complementarity, the systematic error parameters can be decoupled and the solution ambiguity caused by parameter coupling can be avoided. Substituting the Stokes vector determined through this process into Equation (9), two sets of equations can be obtained (in the formula, A1, B1, C1, and D1 correspond to the 0° linearly polarized light input, while A2, B2, C2, and D2 correspond to the 45° linearly polarized light input):

(10) $$\left\{\begin{array}{c} A^{1}=1+\frac{1}{2}(1-\sin\delta )\cos2\alpha \\ B^{1}=\cos\delta \sin2\alpha \\ C^{1}=\frac{1}{2}(1+\sin\delta )\cos(2\alpha -4\Delta \theta )\\ D^{1}=\frac{1}{2}(1+\sin\delta )\sin(2\alpha -4\Delta \theta ) \end{array}\right.$$

(11) $$\left\{\begin{array}{c} A^{2}=1+\frac{1}{2}(1-\sin\delta )\sin2\alpha \\ B^{2}=-\cos\delta \cos2\alpha \\ C^{2}=\frac{1}{2}(1+\sin\delta )\sin(2\alpha -4\Delta \theta )\\ D^{2}=-\frac{1}{2}(1+\sin\delta )\cos(2\alpha -4\Delta \theta ) \end{array}\right.$$

By simultaneously solving the two groups of corresponding equations above, a formula for the calculating the mean error can be derived as

(12) $$\left\{\begin{array}{c} \alpha =\frac{1}{2}\arctan\left(-\frac{B^{1}}{B^{2}}\right)\\ \delta =\arcsin\left(\frac{\sqrt{{\left(C^{1}\right)}^{2}+{\left(D^{1}\right)}^{2}}-\sqrt{{\left(C^{2}\right)}^{2}+{\left(D^{2}\right)}^{2}}}{\sqrt{{\left(C^{1}\right)}^{2}+{\left(D^{1}\right)}^{2}}+\sqrt{{\left(C^{2}\right)}^{2}+{\left(D^{2}\right)}^{2}}}\right)\\ \Delta \theta =\frac{2\alpha -\arctan\left(\frac{D^{1}C^{2}-C^{1}D^{2}}{C^{1}C^{2}+D^{1}D^{2}}\right)}{4} \end{array}\right.$$

From this, the error values of each polarization device can be obtained. Based on these, compensation is carried out for each polarization device to improve their measurement accuracy.

## 3. Simulation Calculation and Analysis

In order to analyze the calibration accuracy of this method, 86 points were recorded in one cycle when the angle error of the polarizer’s light transmission axis was α=0.5°, the angle error of the 1/4 wave plate’s fast axis was ∆θ=0.5°, the phase delay value error was δ=1°, the turntable’s rotation positioning deviation during the calibration process was 1’, and the signal’s mean value was 1% of the random noise of the system. The process of calibrating the device parameters was simulated, and the parameters were calculated 100 times. The simulation results are shown in Table 1.

The device parameter error values obtained through simulation calculation were α=0.5193°,∆θ=0.5103°, and δ=−0.9856°. Since we needed to rotate the polarizer to 0° or 45° during the calibration process, an angle error occurred in the polarizer’s light transmission axis during this step, resulting in deviations between the calculated device parameter error values and the assumed values of α=0.5°, $∆\theta_{0}=0.5{}^{\circ},\;and\;\delta =1{}^{\circ}$, which were 0.0193°, 0.0103°, and 0.0144°, respectively. This deviation is the calibrated device parameter error. That is, through the device parameter calibration process, the errors reduced the original values of $\alpha =0.5{}^{\circ},∆\theta_{0}=0.5{}^{\circ},\;and\;\delta =1{}^{\circ}$ to $\alpha =0.0193{}^{\circ},∆\theta_{0}=0.0103{}^{\circ},\;and\;\delta =0.0144{}^{\circ}$.

**Figure 3:**
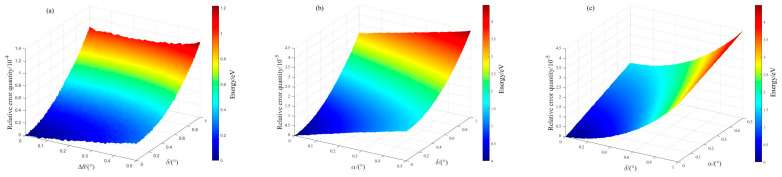
Diagrams of the parameter error calibration results from the simulation calculation: (**a**) the relative error distribution between the simulated calculated values and assumed values of the 1/4 wave plate’s fast-axis angle error, ∆θ; (**b**) the relative error distribution between the simulated calculated values and assumed values of the polarizer’s light-transmission–axis angle error, α; (**c**) the relative error distribution between the simulated calculated values and assumed values of the 1/4 wave plate’s phase delay value error, δ.

Figure 3 presents graphs showing the parameter error calibration results from the simulation calculation. Among them, Figure 3a presents the relative error distribution between the simulated calculated values and assumed values of the 1/4 wave plate’s fast-axis angle error, ∆θ, and Figure 3b presents the relative error distribution between the simulated calculated values and assumed values of the polarizer’s light-transmission–axis angle error, α. Figure 3c presents the relative error distribution between the simulated calculated values and assumed values of the 1/4 wave plate’s phase delay value error, δ. It can be seen from the figure that as the parameter error increased, the relative error obtained from the simulation calculation increased accordingly. In Figure 3a, the *x*-axis represents the assumed fast-axis angle error, the y-axis denotes the assumed phase retardation error, and the *z*-axis shows the simulated relative error resulting from their combined influence.

**Figure 4:**
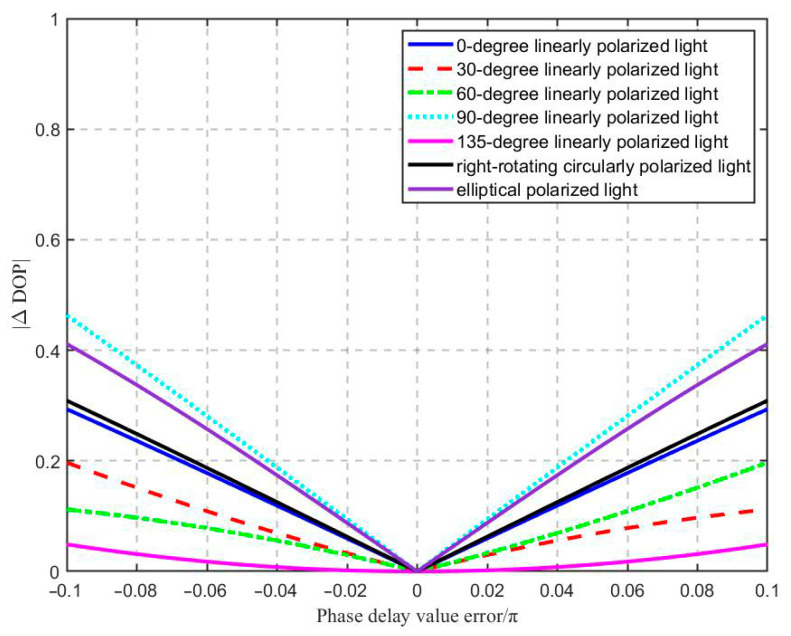
The influence of the phase delay error of the wave plate on DOP.

Figure 4 shows the measurement error values of the degree of polarization (DOP), |DOP|, obtained through simulation calculation under different wave plate phase delay errors and polarization states of the light to be measured. It can be observed from the figure that the |DOP| was the largest when 90° linearly polarized light was to be measured. The larger the wave plate’s phase delay error value, the greater the impact on the DOP measurement result. This conclusion is consistent with previously simulated results of parameter error calibration, in which the relative error obtained from the simulation calculation increased as the parameter error increased.

The measurement accuracy of the polarization detection device before and after calibration was further analyzed. Taking the horizontally polarized light and 45-degree linearly polarized light as the measurement objects, simulation calculations were carried out. The simulation results are shown in Table 2.

The simulation results show that after the calibration of the device parameters, the error of the horizontally polarized light’s polarization degree, P, was reduced from an original value of −2.76% to −0.18%, and the normalized total root mean square deviation, ΔS, was reduced from an original value of 3.24% to 0.26%. The polarization degree error of the 45° linearly polarized light decreased from −2.54% to −0.22%, and the normalized total root mean square deviation decreased from 3.27% to 0.27%. The measurement accuracy of the polarization detection device significantly improved after calibration.

## 4. Experiments and Analysis of Results

### 4.1. Experimental System

**Figure 5:**
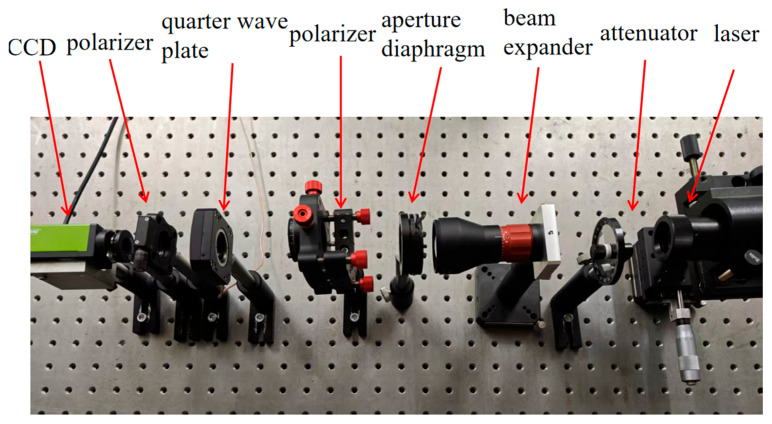
Schematic diagram of parameter calibration for polarization detection device.

Since the measurement of a Stokes vector using the rotating wave plate method is applicable to full-band polarized light, a 632.8 nm wavelength was selected here for experiments investigating the feasibility of measurement and calibration. A schematic diagram of the parameter calibration of the polarization detection device is shown in Figure 5. The device comprised a 632.8 nm He-Ne laser, attenuator, beam expander, aperture diaphragm, polarizer, polarization state analyzer, and CCD camera. The polarization state analyzer comprised a rotatable quarter wave plate with a motor and a fixed polarizer. (The quarter wave plate was on the front, and the polarizer was on the back.)

The laser, beam expander, aperture diaphragm, and linear polarizer were used to generate the linearly polarized light to be measured. The 1/4 wave plate, electric turntable, and polarizing prism comprised the measurement system, and the CCD was used to obtain the light intensity data. Calibration light sources at 0° and 45° were generated by rotating the linear polarizer. The polarization degree of the light produced by the linear polarizer was 99%, and its extinction ratio was better than 1 × 10^−5^. The phase delay accuracy of the zero-level 1/4 wave plate was λ/300. The deviation generator and detector were both fixed on the mobile turntable, the rotational accuracy of which was 1′. The experiment utilized the Zhongchuang SPZ-24-15 (Produced by Wuhan Zhongchuang Vibration Equipment Co., Ltd., a company based in Wuhan, China) precision damping vibration isolation optical platform. This platform boasts specifications including a flatness of <0.05 mm/m^2^, surface roughness of <0.8 μm, repeatability positioning accuracy of ±0.05 mm, and amplitude of <5 μm. Equipped with a unique horizontal adjustment mechanism, it facilitates easy leveling and ensures high stability. The CCD camera employed, a GO-8105M-5GE-UV (JAI) model, features an 8-bit quantization depth, a signal-to-noise ratio exceeding 60 dB, and a pixel size of 2.74 μm.

**Figure 6:**
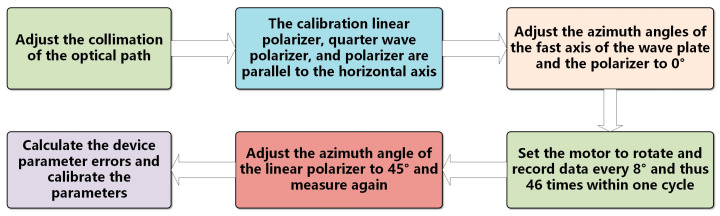
Experimental flowchart.

To avoid the influence of external light source noise, the entire experiment was conducted in a dark environment. The azimuth angle of the polarizer’s light transmission axis was set as the horizontal reference axis of the experimental system, and the azimuth angle of the 1/4 wave plate’s fast axis was determined based on this. The effective selection of sampling points during Fourier analysis plays a critical role in ensuring the desired calculation accuracy and system detection rate. Consequently, to achieve a compromise between the polarization detection error and detection speed and accommodate practical engineering considerations, the parameter measurement employed 46 equally spaced sampling points over one complete rotation of the wave plate. We adjusted the polarizer so that its light transmission axis was parallel to the horizontal axis, thereby generating horizontally polarized light. We adjusted the fast axis of the wave plate to 0°, set the motor to rotate, and recorded data every 8° and thus 46 times within one cycle, generating 46 different polarization states. After that, the linear polarizer was rotated 45° for another measurement. A pupil image was collected at each rotation angle, and the parameter errors of the polarization device and Stokes parameters of the linearly polarized light before and after error compensation were obtained through data processing and calculation. A flowchart of the experimental process is shown in Figure 6.

### 4.2. Analysis of Results

**Figure 7:**
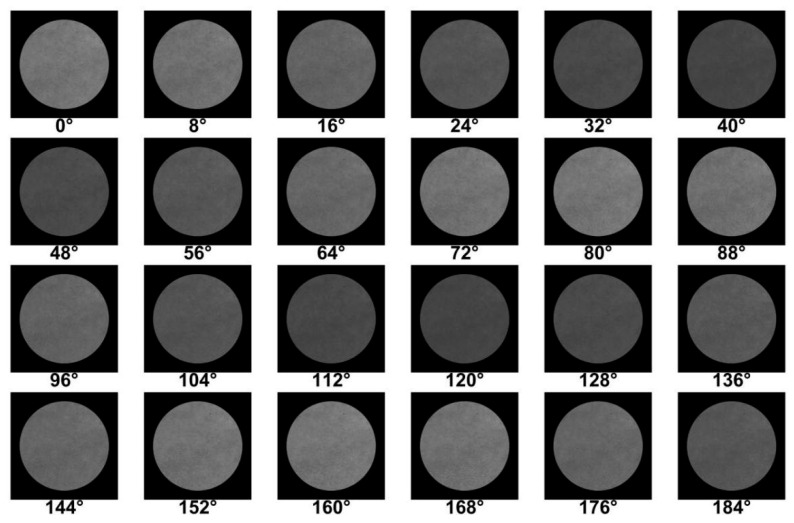
The pupil images taken by the CCD camera.

For each measurement, 46 pupil images were taken using the CCD camera. The first 24 images (from 0° to 184°) are presented in Figure 7, with each measuring 2856 pixels × 2848 pixels. Since the environment in the experimental system was not ideal, the fluctuation of the light source caused an uneven light intensity in the image. In order to reduce the influence of the uneven light intensity on the error calibration results, the images were processed to obtain the average light intensity of each image. Then, all the data were normalized. Through calculation, the angle error of the polarizer’s light transmission axis of was obtained as α=0.5183, the azimuth error of the 1/4 wave plate’s fast axis was obtained as ∆θ = 0.4317°, and the phase delay error was obtained as δ = 1.3182°. This is consistent with the phase delay value accuracy of λ/300 provided by the manufacturer.

**Figure 8:**
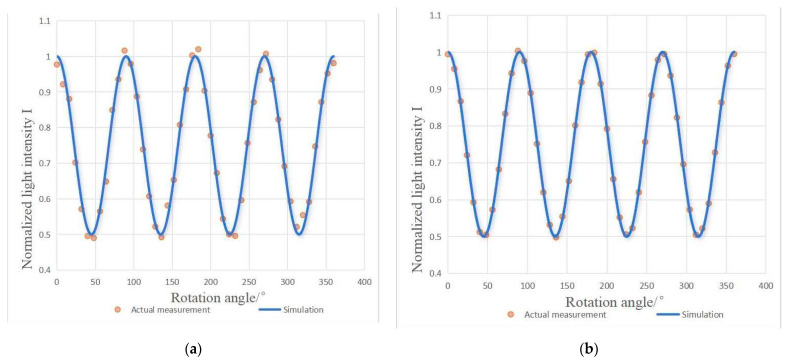
Measurement result comparison chart: (**a**) measurement data before calibration; (**b**) calibrated measurement data.

When the input light was the horizontally polarized standard reference light, its standard value was [1,1,0,0]^T^. The measurement data are shown in the curves in Figure 8, where the left graph (a) represents the measurement data before calibration, and the right graph (b) represents it after calibration. Before calibration, there was a significant difference between the measured data points (orange dots) and the simulated curve (blue solid line), and the fitting degree was low for some wave crests and troughs, indicating that systematic errors (such as the angle of the wave plate, the error of the polarizer, etc.) caused the measured values to deviate from the theoretical model. After calibration, the measured points almost coincided with the simulated curve, and the fitting consistency was significantly improved. It was thus proven that error calibration and compensation effectively corrected the system deviations, making the measurement closer to the theoretical expectations.

Based on this data, a comparison of the measurement results for the input optical Stokes parameters and device parameters before and after calibration is shown in Table 3.

Before the calibration of the device parameters, due to the influence of the angle error of the polarizer’s light transmission axis, the azimuth error of the wave plate’s fast axis, and the phase delay value error, the degree of polarization deviated from the standard value by −3.59%, and the parameters deviated from their standard values by −3.64%, 2.86%, and 1.37%, respectively, with the total root mean square deviation, ΔS, reaching 4.83%. Through the calibration of the device parameters, the deviation of the polarization degree from the standard value was reduced to −0.42%, and the deviations of the parameters from their standard values were reduced to −0.42%, 0.16%, and 0.1%, respectively. The total root mean square deviation, ΔS, was reduced to 0.46%. The measurement accuracy of the polarization detection device significantly improved after calibration.

## 5. Discussion

This study focused on a full-parameter calibration method for rotating wave plates using dual beams, aiming to overcome the inherent limitations of traditional calibration methods in terms of their error coverage and need for accuracy improvements. By constructing an error-transfer analytical model and relying on dual standard polarized light sources (0° and 45° linearly polarized light) for technical support, an innovative “dual standard polarized light calibration” strategy was established to tackle three core error sources: the transmission-axis angle of the polarizer, the fast-axis angle of the wave plate, and the phase delay of the wave plate. This enabled us to achieve the collaborative calibration of multi-parameter errors in the polarization measurement system.

The experimental data show that after calibration using this method, the calibration error of the Stokes parameters decreased from an initial value of 4.83% to within 0.46%. Compared with the traditional calibration methods, this strategy precisely constructs a quantitative mapping relationship between the error and measurement response and relies on an error inversion algorithm to address the device parameter error, significantly improving the calibration accuracy of the polarization detection device. Regarding the level of error coverage, the errors of the three core quantitative parameters are considered simultaneously, overcoming the limitation of single-error correction in some existing schemes (such as methods only correcting the fast-axis angle error of the wave plate and the phase delay value error). Considering these parameters simultaneously is more suited to the working conditions of multi-error coupling in actual polarization measurement scenarios, effectively making up for the incomplete error consideration in traditional methods.

Compared with the achievements of research on polarization measurement calibration technology at home and abroad, this method has significant differentiated advantages. Although foreign polarization measurement technology was developed earlier and is relatively technically mature, in the case of full-parameter error collaborative calibration, it is not suitably adaptable to domestic polarization detection devices. This makes it difficult to meet the precise calibration requirements of domestic equipment. Some domestic studies (such as those focusing on the error correction of a single wave plate or deviation detector) have had problems such as incomplete error coverage and limited calibration accuracy and failed to construct a multi-error coupling calibration mechanism. The method developed in this study achieves the precise calibration of multiple core parameters of a rotating wave plate measurement system. Moreover, it simplifies the operational process using a dual standard polarized light strategy, achieving both high precision and high efficiency. However, while we preliminarily calibrated the polarization detection device parameter errors using the proposed method in a simulation and experiment, in practical applications, errors are typically coupled. Based on the preliminary calibration results, we are currently constructing an error model for the entire lithography process, systematically considering the interference from dynamic scenarios and environmental factors.

In the field of advanced integrated circuit lithography, the polarization state stability of extreme ultraviolet (EUV) [29] lithography light sources directly affects the accuracy of nanoscale pattern transfer. Future research could further optimize the algorithm for polarization device parameter calibration. In response to the requirements for polarized pupil filtering technology in photolithography exposure systems, a high-precision wave plate calibration scheme suitable for use with an EUV light source could be developed. For instance, by enhancing the calibration accuracy of the wave plate’s phase delay error to within 0.1° and using this dual-beam full-parameter calibration strategy, the uniformity of the lithography light source’s polarization state could be controlled within a 6° polar angle range, thereby optimizing the line width roughness (LWR) [30] and critical dimensional uniformity (CDU) [31,32] of the photoresistor after exposure. Furthermore, a machine learning inversion model could be used to construct an algorithm that predicts the error due to polarization state coupling, which is caused by multi-beam interference in lithography. This would provide calibration support for the use of polarization resolution enhancement technology (RET) [33,34] in immersion lithography.

## 6. Conclusions

This study systematically established and analyzed a parameter calibration technique for polarization detection devices based on a dual-beam rotating wave plate method and performed systematic theoretical analysis and experimental verification. The method allowed for the calibration of three quantitative parameter errors, namely the angle error of the polarizing prism’s light transmission axis, the angle error of the wave plate’s fast axis, and the wave plate’s phase delay error. Specifically, 0°([1,1,0,0]^T^) and 45°([1,0,1,0]^T^) linearly polarized standard light sources were used to calibrate the three errors, and simulation analysis was carried out. The simulation and experimental results show that this method can effectively reduce the influence of parameter errors on measurement accuracy. After the calibration of the polarization detection device parameters, the device’s measurement accuracy significantly improved. The error of the polarization degree, P, of the horizontally linearly polarized light decreased from −3.59% to −0.42%, and the standardized total root mean square deviation, ΔS, decreased from 4.83% to 0.46%, providing a reliable method for the fabrication of polarization sensors.

## Figures and Tables

**Table 1:** The device parameter error values obtained through simulation.

Error Factor	Mean Value/°	Standard Deviation/°	Deviation/%
α	0.5193	0.0673	1.93
∆θ	0.5103	0.0694	1.03
δ	0.9856	0.0762	−1.44

**Table 2:** Comparison of simulation results before and after parameter calibration.

Polarized Light		Before Calibration		After Calibration	
Type	Parameter	Value	Error/%	Value	Error/%
Horizontally linearly polarized light	P	0.9724	−2.76	0.9982	−0.18
	$S_{10}$	0.9723	−2.77	0.9982	−0.18
	$S_{20}$	0.0168	1.68	0.0018	0.18
	$S_{30}$	0.001	0.01	0.0001	0.01
	∆S	-	3.24	-	0.26
45-degree linearly polarized light	P	0.9746	−2.54	0.9978	−0.22
	$S_{10}$	0.0203	2.03	0.0013	0.13
	$S_{20}$	0.9744	−2.56	0.9978	−0.22
	$S_{30}$	0.001	0.1	0.001	0.1
	∆S	-	3.27	-	0.27

**Table 3:** Comparison of measurement results before and after parameter calibration.

Polarized Light		Before Calibration		After Calibration	
Type	Parameter	Value	Error/%	Value	Error/%
Horizontally linearly polarized light	P	0.9641	−3.59	0.9958	−0.42
	$S_{10}$	0.9636	−3.64	0.9958	−0.42
	$S_{20}$	0.0286	2.86	0.0016	0.16
	$S_{30}$	0.0137	1.37	0.001	0.1
	∆S	-	4.83	-	0.46

## Data Availability

Due to particular reasons, the data from this project are temporarily not being made public.
